# Two decades of fumigation data from the Soybean Free Air Concentration Enrichment facility

**DOI:** 10.1038/s41597-023-02118-x

**Published:** 2023-04-20

**Authors:** Elise Kole Aspray, Timothy A. Mies, Jesse A. McGrath, Christopher M. Montes, Bradley Dalsing, Kannan K. Puthuval, Andrew Whetten, Jelena Herriott, Shuai Li, Carl J. Bernacchi, Evan H. DeLucia, Andrew D. B. Leakey, Stephen P. Long, Justin M. McGrath, Franco Miglietta, Donald R. Ort, Elizabeth A. Ainsworth

**Affiliations:** 1 Global Change and Photosynthesis Research Unit, United States Department of Agriculture, Agricultural Research Service, Urbana, IL 61801 USA; 2 Department of Plant Biology, University of Illinois at Urbana-Champaign, 505 S. Goodwin Ave, Urbana, IL 61801 USA; 3 Institute of Genomic Biology, University of Illinois at Urbana-Champaign, 1206 W. Gregory Drive, Urbana, IL 61801 USA; 4 Department of Crop Sciences, University of Illinois at Urbana-Champaign, 1102 S. Goodwin Ave, Urbana, IL 61801 USA; 5 Department of Mathematical Sciences, University of Wisconsin-Milwaukee, 2200 E Kenwood Blvd, Milwaukee, WI 53211 USA; 6 Department of Agriculture and Applied Sciences, Langston University, 701 Sammy Davis Jr. Drive, Langston, OK 73050 USA; 7 National Research Council of Italy, Institute for Bioeconomy (CNR IBE), Florence, Italy

**Keywords:** Environmental sciences, Plant sciences

## Abstract

The Soybean Free Air Concentration Enrichment (SoyFACE) facility is the longest running open-air carbon dioxide and ozone enrichment facility in the world. For over two decades, soybean, maize, and other crops have been exposed to the elevated carbon dioxide and ozone concentrations anticipated for late this century. The facility, located in East Central Illinois, USA, exposes crops to different atmospheric concentrations in replicated octagonal ~280 m^2^ Free Air Concentration Enrichment (FACE) treatment plots. Each FACE plot is paired with an untreated control (ambient) plot. The experiment provides important ground truth data for predicting future crop productivity. Fumigation data from SoyFACE were collected every four seconds throughout each growing season for over two decades. Here, we organize, quality control, and collate 20 years of data to facilitate trend analysis and crop modeling efforts. This paper provides the rationale for and a description of the SoyFACE experiments, along with a summary of the fumigation data and collation process, weather and ambient data collection procedures, and explanations of air pollution metrics and calculations.

## Background & Summary

Prior to the Industrial Revolution, the concentration of carbon dioxide ([CO_2_]) in the atmosphere did not exceed 280 parts per million (ppm) for at least 800,000 years^1^. The global monthly mean for ambient [CO_2_] was 416 ppm in October of 2022, a ~50% increase since industrialization and a ~20% increase since 1980 [https://gml.noaa.gov/ccgg/trends/global.html]. Atmospheric [CO_2_] is continuing to rise at an unprecedented rate and without significant emissions reductions, the concentrations will reach 700 to 1100 ppm by 2100^2^. In addition to CO_2_, the tropospheric concentration of ozone ([O_3_]) has also increased since the Industrial Revolution. Although O_3_ in the stratosphere, which is about eight kilometers above the Earth’s surface, provides a useful barrier for ultraviolet radiation, O_3_ in the surface tropospheric layer is a toxic pollutant. The ~40% increase in tropospheric [O_3_] since industrialization^3^ has caused harm to humans, animals, and plants^4^. These increases in CO_2_ and O_3_ concentrations have already directly impacted plants^5,6^, and future atmospheric increases will only intensify impacts on agriculture.

For over a century, scientists have tried to understand the effects of atmospheric change on agriculture^6–10^. To this end, researchers have experimented with different approaches to alter greenhouse gas concentrations around plants. Controlled environmental enclosures, including growth chambers, allow for relatively precise control of light, humidity, temperature, and atmospheric composition, enabling scientists to mechanistically test how CO_2_ or O_3_ affect plant function while all other conditions are held constant. However, growth chambers are typically small which limits the number of individual plants that can be examined, and there are often inconspicuous differences in supposedly identical chambers^11^. Also, in addition to enriching the air with CO_2_ and O_3_, chamber experiments can have unwanted impacts on other aspects of the environment surrounding the plant^12^. For example, plants in chamber studies are grown in pots which can restrict root growth, altering the plant response to elevated [CO_2_]^13,14^. Greenhouses and naturally sunlit outdoor growth chambers are an alternative option to indoor chambers and can be used to study plants in natural environments^9^. Because the sides of outdoor chambers typically are made of acrylic plastic, they can partially block radiation and potentially increase temperature and humidity. Outdoor chambers can also increase [CO_2_] at the plant canopy level if the fumigation is released from the bottom of the chamber^9^. Additionally, open-top chambers (OTCs) shelter vegetation from the wind and force air upwards through the canopy, which alters the natural atmospheric coupling^12^. Thus, although outdoor chambers use aspects of the natural environment to operate, they can also modify the surrounding environment in undesirable ways similar to indoor chambers. These environmental and atmospheric impacts make the results of chamber studies less comparable to the natural response of plants to climate change, which is a clear limitation of both indoor and outdoor growth chambers.

Free Air Concentration Enrichment (FACE) facilities were developed as a ‘real-world’ approach to understanding how plants respond to altered atmospheres in fully open-air conditions^9,10,15,16^. An important benefit of the FACE approach is the ability to study the interaction of multiple atmospheric variables with elevated [CO_2_] and [O_3_], and the effects of these interactions on plants in nature^17^. Unlike growth chambers which are restricted to only 1 to 15 m^2^ in size, FACE plots can range in size from 100 to 300 m^2^, allowing for experiments that are larger in scope and more varied than OTC experiments^18^. A typical FACE plot consists of a circular or octagonal array of pipes that release CO_2_ or air enriched with CO_2_ or O_3_ at the canopy surface for small stature vegetation, or at varying levels from the ground to the top of the canopy for larger stature vegetation^10^. The [CO_2_] or [O_3_] is measured at the center of the plot, along with wind speed and direction. A computer-controlled system adjusts flow rates to maintain the target gas concentration in the center of the plot. While different FACE facilities have used vertical or horizontal release pipes with or without blower systems to mix the air, the general design has been used to study the response of natural and managed ecosystems to elevated [CO_2_] and/or [O_3_] around the world^19,20^.

**Fig. 1:**
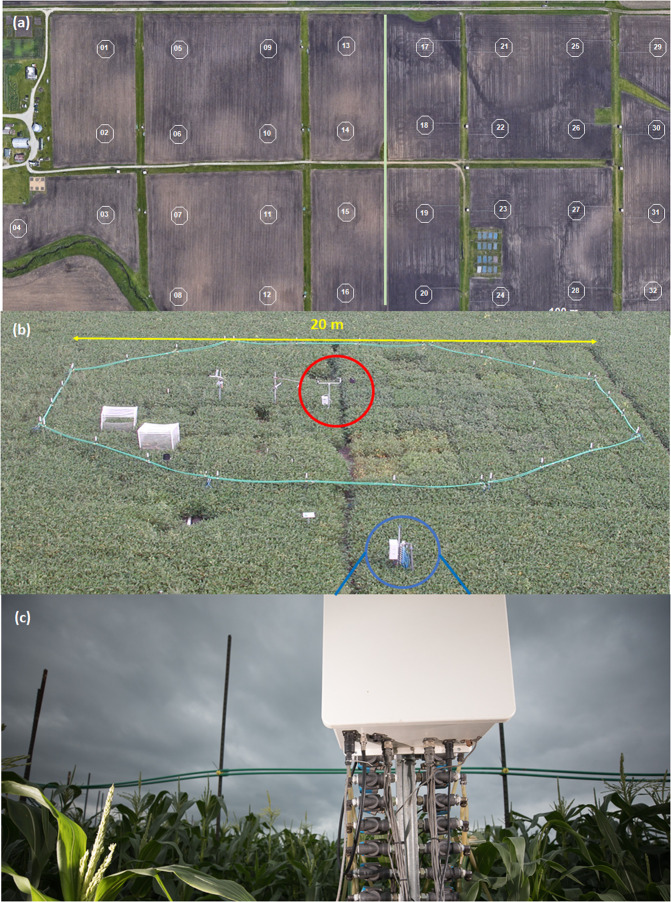
(**a**) Aerial view of the SoyFACE experiment (from Google Earth) with the 32 octagonal CO_2_, O_3_, combination, or ambient plots. Half of the field (i.e., plots 1–16 or 17–32; separated by a white line in the image) is planted in soybean and the other half planted in maize with crops rotated on an annual basis. (**b**) Image of a single SoyFACE treatment plot from the fumigation experiments. The wind sensor is circled in red at the center of the octagonal plot. The manifold (gas delivery system) that delivers the CO_2_ and O_3_ used in the experiments is circled in blue outside of the plot. Photo credit: Andrew Leakey. (**c**) Close-up image of the manifold and outgoing pipes outside of a SoyFACE treatment plot. These outgoing pipes deliver the CO_2_ and O_3_ gases to the green pipes along the sides of the treatment plots, from which the gases are released. Photo credit: Scott Gable.

The Soybean Free Air Concentration Enrichment (SoyFACE) facility in Illinois was first developed in 2001 and has been operational for over two decades, making it the longest-running FACE facility (Fig. 1). SoyFACE contains replicated elevated [CO_2_] and elevated [O_3_] plots, along with ambient plots, within a 32 ha farm. Initially, experiments focused on understanding the responses of soybean and maize to elevated [CO_2_] and [O_3_]^21–26^ and later experiments investigated the interaction of CO_2_ with temperature^27,28^ and drought stress^29–31^. More recently, the facility has been used to study genetic variation in crop responses to atmospheric change^32–34^ and genetically engineered adaptation to rising [CO_2_] and temperature^35,36^. During the growing season, ambient [CO_2_] and [O_3_] and FACE [CO_2_] and [O_3_] data are collected every four seconds along with the wind speed and wind direction. These data are averaged to produce 1-minute fumigation^37^ (File 9) and ambient data files, which can be used to calculate fumigation efficiency statistics and hourly, daily, monthly, and seasonal fumigation and ambient data metrics. As the longest running FACE facility in the world, it is fitting that we provide broad access of our data to the scientific community. The abundance of fumigation data from experiments executed over two decades make the SoyFACE fumigation data particularly useful for modeling the impacts of rising CO_2_ and O_3_ on crop physiology and agronomy, as well as ecosystem function.

## Methods

### Field site & experimental design

The 32 ha SoyFACE farm is located on the south side of the University of Illinois Urbana-Champaign campus (228 meters above sea level; 40° 02′ North, 88° 14′ West). Each year, approximately half of the field is planted with soybean (*Glycine max*) and half with maize (*Zea mays*). The maize crop is fertilized with ~200 kg N ha^−1^ and the soybean crop is not fertilized. Lime has been periodically applied to the crops over the past 20 years, with pre- and post-emergent herbicides applied to the crops per common practice in the region. The two predominant soil types at the farm are Drummer silty clay loam and Flanagan silt loam. Within the 32 ha field, there are fixed locations for 32 octagonal plots. Soybean and maize are each grown over roughly half of the facility in a given year, and rotate positions in successive years. Each FACE plot is 20 meters in diameter with an area of ~280 m^2^ (Fig. 1a,b). In a given year, experiments within a specific crop have used 8–16 plots in randomized complete block designs. Most experiments have been designed with replication at the plot level of n = 4 (Tables 1, 2). In some cases, split-plot treatments of drought or temperature have also been applied^27,38^. The crops are typically planted between mid-May and mid-June, with fumigation starting within two weeks of the planting date and crop harvests occurring in September and October.

**Table 1:** SoyFACE experiment details, 2001–2010. Each year consists of 1–5 distinct experiments.

Year	Treatment	Species	Plots	Planting Date	Treating Date	Harvest Date	Gas Setpoint
2001	CO_2_	soybean	3,5,14,15	5/23/2001	6/3/2001	10/18/2001	550
2002	CO_2_	soybean	20,21,28,29	6/1/2002	6/9/2002	10/16/2002	550
2002	ozone	soybean	19,22,27,30	6/1/2002	6/25/2002	10/16/2002	1.5 × ambient
2002	CO_2_	maize	3,5,14,15	5/30/2002	6/6/2002	10/10/2002	550
2003	CO_2_	soybean	3,5,14,15	5/27/2003	6/7/2003	10/15/2003	550
2003	ozone	soybean	2,7,12,13	5/27/2003	6/17/2003	10/15/2003	1.5 × ambient
2003	CO_2 + _ozone	soybean	6,8,9,16	5/27/2003	6/17/2003	10/15/2003	550, 1.5 × ambient
2004	CO_2_	soybean	20,21,28,29	5/28/2004	6/5/2004	10/5-7/2004	550
2004	ozone	soybean	19,22,27,30	5/28/2004	6/17/2004	10/5-7/2004	1.5 × ambient
2004	CO_2 + _ozone	soybean	18,23,26,31	5/28/2004	6/17/2004	10/5-7/2004	550, 1.5 × ambient
2004	CO_2_	maize	3,5,14,15	4/29/2004	5/9/2004	9/10/2004	550
2005	CO_2_	soybean	3,5,14,15	5/25/2005	5/31/2005	10/26/2005	550
2005	ozone	soybean	2,7,12,13	5/25/2005	6/7/2005	10/26/2005	1.5 × ambient
2005	CO_2 + _ozone	soybean	6,8,9,16	5/25/2005	6/7/2005	10/26/2005	550, 1.5 × ambient
2006	CO_2_	soybean	20,21,28,29	5/26/2006	5/31/2006	10/5/2006	550
2006	ozone	soybean	19,22,27,30	5/26/2006	6/6/2006	10/5/2006	1.5 × ambient
2006	CO_2_ + ozone	soybean	18,23,26,31	5/26/2006	6/6/2006	10/5/2006	550, 1.5 × ambient
2007	CO_2_	soybean	3,5,14,15	5/22/2007	5/29/2007	10/3/2007	550
2007	ozone	soybean	2,7,12,13	5/22/2007	6/4/2007	10/3/2007	2 × ambient
2007	CO_2_ + ozone	soybean	6,8,9,16	5/22/2007	6/4/2007	10/3/2007	550, 2 × ambient
2008	CO_2_	soybean	20,21,28,29	6/17/2008	6/26/2008	10/29/2008	550
2008	ozone	soybean	19,22,27,30	6/17/2008	7/11/2008	10/29/2008	2 × ambient
2008	CO_2_ + ozone	soybean	18,23,26,31	6/17/2008	7/11/2008	10/29/2008	550, 2 × ambient
2008	CO_2_	maize	3,5,14,15	5/29/2008	6/10/2008	10/1/2008	550
2009	CO_2_	soybean	3,5,14,15	6/9/2009	6/19/2009	10/20/2009	585
2009	ozone	soybean	2,6,7,8,9 12,13,16	6/9/2009	6/29/2009	10/20/2009	40,55,70,85,110,130,160,200
2010	CO_2_	maize	3,5,14,15	4/28/2010	5/10/2010	9/14/2010	585
2010	CO_2_	soybean	20,21,28,29	5/27/2010	6/10/2010	9/30/2010	585
2010	ozone	soybean	18,19,22,23 26,27,30,31	5/27/2010	6/6/2010	9/30/2010	55,70,85,110,130,150,170,190

Each experiment consists of either 4 or 8 treatment plots, a fumigation treatment of CO_2_, O_3_, or both CO_2_ and O_3_, a single crop species, planting, treatment, and harvest dates, and the CO_2_ or O_3_ setpoint, measured in parts per million (ppm) for CO_2_ and parts per billion (ppb) for O_3_.

**Table 2:** SoyFACE experiment details, 2011-2021.

2011	CO_2_	soybean	3,5,14,15	6/8/2011	6/15/2011	10/4/2011	590
2011	ozone	soybean	2,6,7,8	6/8/2011	6/21/2011	10/4/2011	100
2011	ozone	soybean	9,12,13,16	6/8/2011	6/21/2011	10/4/2011	100
2012	CO_2_	soybean	20,21,28,29	5/16/2012	5/25/2012	10/15/2012	590
2012	ozone	soybean	18,19,22,23 26,27,30,31	5/15/2012	5/29/2012	9/29/2012	100/110
2013	CO_2_	soybean	3,5,14,15	6/7/2013	6/14/2013	11/1/2013	600
2013	ozone	soybean	2,6,7,8	6/6/2013	7/7/2013	9/29/2013	100
2013	ozone	soybean	9,12,13,16	6/6/2013	7/7/2013	9/29/2013	100
2013	ozone	maize	18,19,22,23	5/16/2013	6/8/2013	*No yield measure	100
2013	ozone	maize	26,27,30,31	5/16/2013	6/8/2013	*No yield measure	100
2014	ozone	maize	2,3,6,7	5/19/2014	6/7/2014	9/5-10/20/2014	100
2014	ozone	maize	9,12,13,16	5/19/2014	6/7/2014	9/5-10/20/2014	100
2014	CO_2_	soybean	20,21,28,29	6/18/2014	6/29/2014	11/1/2014	600
2015	CO_2_	soybean	3,5,14,15	6/5/2015	6/7/2015	10/13/2015	600
2015	ozone	maize	18,19,22,23	5/19/2015	6/1/2015	9/22-10/21/2015	100
2015	ozone	maize	26,27,30,31	5/19/2015	6/1/2015	9/22-10/21/2015	100
2016	ozone	maize	2,3,6,7	5/24/2016	6/2/2016	9/15-10/20/2016	100
2016	ozone	maize	9,12,13,16	5/24/2016	6/2/2016	9/15-10/20/2016	100
2016	CO_2_	soybean	20,21,28,29	6/6/2016	6/11/2016	10/12/2016	600
2017	CO_2_	soybean	5,6,9,14	5/29/2017	6/25/2017	10/7/2017	600
2017	CO_2_	cassava	3,8,15,16	6/3/2017	6/10/2017	9/30/2017	600
2017	ozone	maize	18,19,22,23	5/17/2017	6/6/2017	9/17-10/27/2017	100
2017	ozone	maize	26,27,30,31	5/17/2017	6/6/2017	9/17-10/27/2017	100
2018	ozone	maize	9,12,13,16	5/13/2008	5/25/2018	9/21/2018	100
2018	CO_2_	cassava	20,21,22,23	5/14/2008	6/4/2018	10/5/2018	600
2018	CO_2_	soybean	26,28,29,31	5/17/2018	6/1/2008	9/28/2018	600
2019	CO_2_	soybean	9,14,15,16	5/31/2019	6/11/2019	10/7/2019	600
2019	ozone	C_4_ grasses	18,22,26,30	5/31/2019	6/12/2019	10/3/2019	100
2020	CO_2_	soybean	20,21,28,29	6/1/2020	6/16/2020	10/12/2020	600
2020	ozone	soybean	18,22,26,30	6/1/2020	6/19/2020	10/12/2020	100
2021	CO_2_	soybean	3,5,6,8	5/6/2021	5/23/2021	9/20/2021	600
2021	ozone	soybean	9,12,13,16	5/6/2021	6/1/2021	9/20/2021	100

Each year consists of 1–5 distinct experiments. Each experiment consists of either 4 or 8 treatment plots, a fumigation treatment of CO_2_ or O_3_, a single crop species, planting, treatment, and harvest dates, and the CO_2_ or O_3_ setpoint, measured in parts per million (ppm) for CO_2_ and parts per billion (ppb) for O_3_.

### Fumigation system

Outside of the six-year period between 2003 and 2008, any given treatment plot was fumigated with only elevated [CO_2_] or elevated [O_3_] treatments (Tables 1, 2). Between 2003 and 2008, some plots were simultaneously fumigated with both CO_2_ and O_3_ in a ‘combined’ treatment that allowed testing of interaction effects when studied in conjunction with the CO_2_-only and O_3_-only plots. Wind speed and [CO_2_] and [O_3_] are measured from the center of each plot (Fig. 1b) and sent to a computer control system in the field, which inputs the measurements into the Proportional Integral Differential (PID) algorithm (Eq. 1). The PID algorithm is a commonly used control process that uses key sensor inputs and setpoints to calculate the output variable. The SoyFACE PID algorithm includes a wind speed component that is not found in other PID algorithms and was developed by Lewin *et al*.^39^. In the case of the SoyFACE experiments, the sensor inputs are the wind speed and current [CO_2_] and [O_3_], the setpoints are the target [CO_2_] and [O_3_], and the output variable is the voltage that should be used for the gas valves in order to maintain the setpoints. The setpoints for [CO_2_] and [O_3_] in the SoyFACE plots for each experiment between 2001 and 2021 are given in Table 1 (2001 through 2010) and Table 2 (2011 through 2021). The process of adjusting the CO_2_ and O_3_ levels is inexact since it is impossible to predict exactly which valve setting should be used to add a specific amount of gas to each plot. However, by using the PID algorithm at frequent intervals, the fumigation system is able to use the valve settings to quickly correct gas level adjustments that are above or below the desired amount. The goal of the SoyFACE system is to maintain the gas levels as closely as possible to the setpoints by continually measuring the current levels, implementing the PID algorithm, and releasing additional gases into the plots as needed.

1 $${G}_{cv}={K}_{p}* \left({G}_{stpt}-{G}_{pv}\right)+{K}_{i}* Int\left({G}_{stpt}-{G}_{pv}\right)+{K}_{d}* Der\left({G}_{stpt}-{G}_{pv}\right)+{K}_{w}* ws$$

The variables in Eq. 1 represent the components of the PID algorithm, as defined below.

1. *G*_*cv*_ (*Control/Output Variable*): The voltage applied to the valve that controls release of CO_2_ or O_3_ into the plot.
2. *G*_*stpt*_ (*Setpoint*): The setpoint of CO_2_ or O_3_ in the plot.
3. *G*_*pv*_ (*Process Variable/Sensor Input*): The current level of CO_2_ or O_3_ in the plot.
4. *ws* (*Wind Speed/Sensor Input*): The current wind speed, measured from the center of the plot.
5. *K*_*p*_: Proportional coefficient; indicates that the control variable should be adjusted proportionally to the error in the system, or how much the process variable differs from the setpoint: *error* = (*G*_*stpt*_ – *G*_*pv*_).
6. *K*_*i*_: Integral coefficient.
7. *K*_*d*_: Differential coefficient.
8. *K*_*w*_: Wind coefficient.
9. *Int()*: Integral function; measures the accumulation of the error over time.
10. *Der()*: Differential function; compensates for sudden changes in the error.

Coefficients *K*_*p*_*, K*_*i*_*, K*_*d*_, and *K*_*w*_ are constant values determined through analysis of iterative test runs of the fumigation process in early SoyFACE experiments. Currently the coefficients are as follows:

**Table:** 

Coefficient	Value for CO_2_ Experiments	Value for O_3_ Experiments
*K*_*p*_	0.0008	0.0016
*K*_*i*_	0.000015	0.00008
*K*_*d*_	0.016	0.016
*K*_*w*_	0.15	0.5

The fumigation system at the SoyFACE farm was initially designed to distribute CO_2_ to the treatment plots. The early SoyFACE model was based upon the system used at a poplar FACE plantation (POPFACE) in Tuscania, Italy. This fumigation system was designed to release pure CO_2_ into the atmosphere at a high velocity through a large number of small regularly spaced air jets to create a shock wave and turbulence at the gas exit point. This enhanced the mixing of CO_2_ into the ambient air and since the jets face outward, the CO_2_ is mixed with the air prior to being carried by the wind back into the plot. This provides a relatively uniform elevated [CO_2_] within the plot^16^.

In addition to the air jet configuration, determining the ideal pressure settings for each step of the CO_2_ fumigation process was critical. To this end, a manually controlled pressure generator with narrow perforations was used to modify the pressure of the CO_2_ gas flow within the pipes in eight steps, from 0.15 MPa to 0.45 MPa. The ability to change the air pressure allowed for better regulation of the CO_2_ flow rate as the gas was transported through an underground HDPE pipeline to horizontal pipes along the sides of the octagonal plots. Additionally, the voltage calculated by the PID algorithm regulated the pressure inside the horizontal pipes and controlled the amount of CO_2_ entering the plots. Following the release of CO_2_, natural wind currents facilitated the distribution of CO_2_ throughout each plot.

**Fig. 2:**
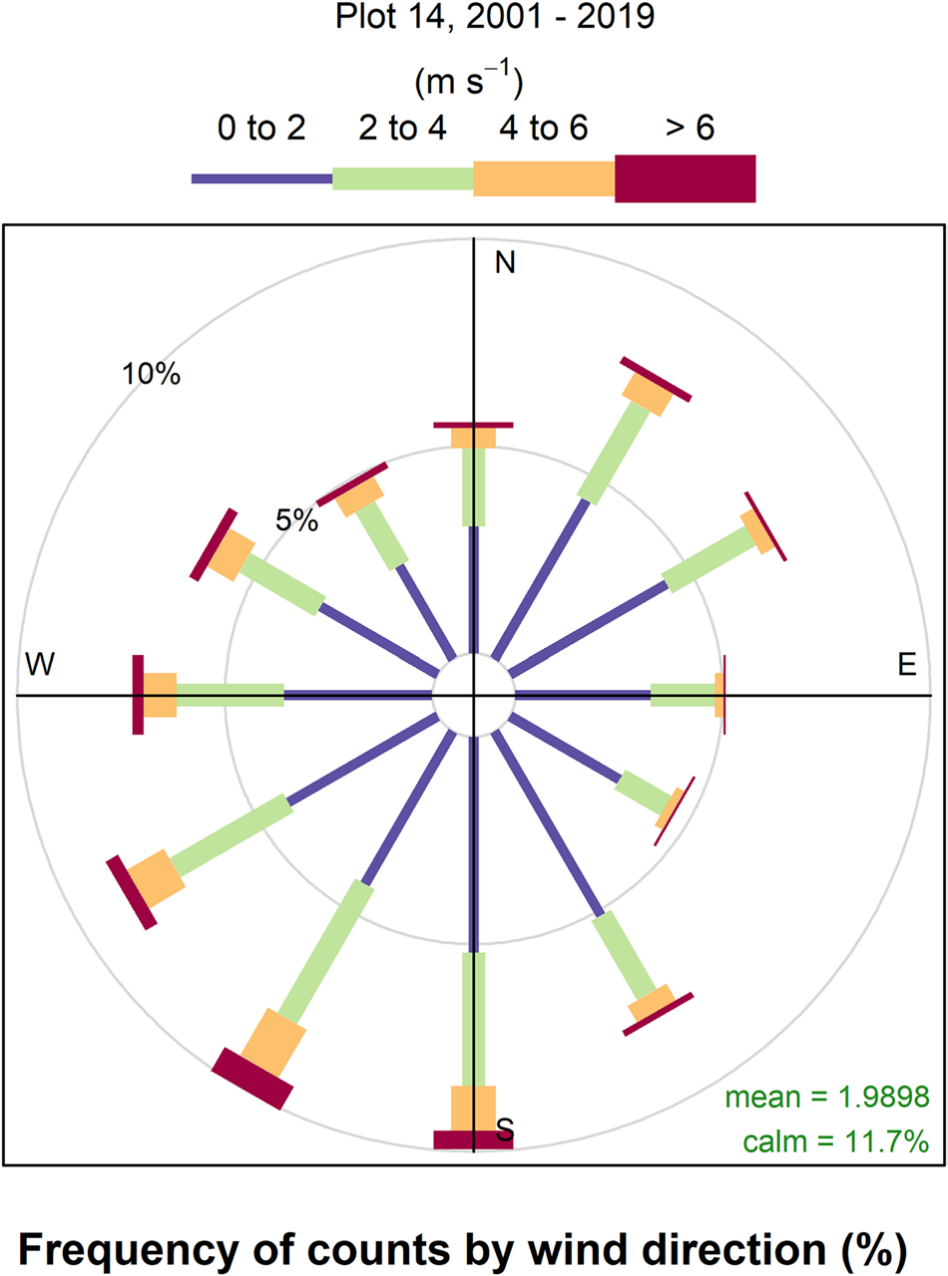
Wind rose plot generated from 1-minute wind speed and direction data collected from Plot 14 between 2001 and 2019. Wind rose plots are polar graphs generated with the *openair* package in R^48^. The radii length of the concentric circles represents the percentage frequency of measurements that have wind speeds between 0 to 2 m/s (blue), 2 to 4 m/s (green), 4 to 6 m/s (orange), and >6 m/s (red). The wind direction ranges include plus or minus 15 degrees from the given direction, starting at 0° on the upper vertical axis (N) and moving clockwise in 30° increments back to 360°. In the lower right corner of the plots, the *mean* refers to the overall mean wind speed for the plot, and the *calm* percentage indicates the percentage of calm observations omitted from the wind rose plot. Following the convention of the National Weather Service, winds with a direction of 0° are considered ‘calm’, while winds with a direction of 360° are assumed to be from the north.

The CO_2_ fumigation system at SoyFACE retains much of the same design of the original POPFACE model. Liquid CO_2_ is stored in a 50-ton vertical tank at the SoyFACE facility and passed through vaporizing equipment to produce gaseous CO_2_, which is delivered to specific locations in the field through underground pipes. CO_2_ is transported to a manifold (gas delivery system; Fig. 1c) outside of the plot and tubing delivers the CO_2_ to the tubes surrounding the treatment plots. CO_2_ is released through a linear flow valve (SMC pressure controllers^16^) and pure CO_2_ is released into the wind through 350 or 500 small air jets placed 15 mm apart and drilled into 8-meter-long pipes that surround the SoyFACE plots (Fig. 1). The flow valves have settings between 0 and 10, with a setting of 0 indicating a completely closed valve and a setting of 10 indicating a completely open valve. The control computer system (formerly Z-World Inc. Model SR9000; currently Rabbit BLS4200 series) calculates the amount of gas that should be released into each plot based on information from wind sensors (R.M. Young Model 12005), CO_2_ analyzers (PP Systems SBA series), and the PID algorithm. The high jet velocity of the CO_2_ gas stream creates rapid dilution with the ambient air^17^. Throughout the past 20 years, as seen in Fig. 2a,b, the mean wind speed varied slightly over the site, and was 2.0 and 1.7 m/s in Plot 14 and Plot 3, respectively. These two plots exemplify the variation in wind speed and direction measured at the site and were used in most of the past 20 years (Tables 1, 2). In the instances when the wind speeds dropped below 0.2 m/s, the CO_2_ fumigation system cycled CO_2_ gas around the plots to maintain the setpoint as closely as possible.

Testing of different aspects of the CO_2_ fumigation system at POPFACE and other FACE sites assisted with the development of the CO_2_ fumigation process used at SoyFACE. However, CO_2_ was not the only greenhouse gas of interest, and the fumigation system was modified to deliver O_3_ treatments in addition to CO_2_. In preparation of the O_3_ fumigation process, liquid oxygen is stored in a 450-liter cryogenic tank at SoyFACE. The liquid O_2_ is passed through vaporizing equipment to produce gaseous O_2_. Then, a generator is used to pass gaseous O_2_ through a high-voltage dielectric field (~6000 volts) inside a generator (PCI-Wedeco Model GA40 prior to 2005; currently Ozonia Ozat Model CFS-3 2 G). The dielectric field forces some of the O_2_ molecules to disassociate and recombine to form O_3_, producing up to 3.5 kg of O_3_ per day. Due to the toxicity of O_3_ and the fact that the generator produces the gas at a low pressure, it must be pressurized and mixed with compressed air before being transported to the SoyFACE plots. This is accomplished with a bypass venturi differential pressure injector (Mazzei Injectors Model 384-X), which forces O_3_ to mix into the higher-pressure air stream. The compressed air stream enters the bypass pressure injector at 90 PSI, O_3_ enters the pressure injector at a low pressure of 8 PSI, and the resulting O_3_-air mixture has a pressure of 35 PSI. The change in pressure allows the O_3_ gas mixture to be delivered from the pressure injector to the manifold. Computer-controlled linear flow valves (PCI-Wedeco Model GA40 prior to 2005; currently Teledyne Hastings Model HCF-302) control the release of O_3_-enriched air into the wind. The concentration of O_3_ in the center of each octagonal plot is monitored with an O_3_ analyzer (Thermo Fisher Scientific Model 49 C/49I), and that information is used to control the setpoint with the PID algorithm as described for CO_2_ fumigation.

The gas concentrations and wind data are transmitted to a central computer located in an onsite trailer for general data storage and performance analysis. The control computer uses the wind direction measurements to control which main sector of the octagonal treatment plot releases CO_2_ or O_3_-enriched air, with the two neighboring octagon sectors releasing a smaller amount of CO_2_ or O_3_. Since the three fumigation entry sectors are most directly upwind, following the high-pressure valve release, the gases distribute evenly throughout the octagon and dilute to the background gas concentrations within ~100 meters of the plot.

### Data collection & processing methods

**Fig. 3:**
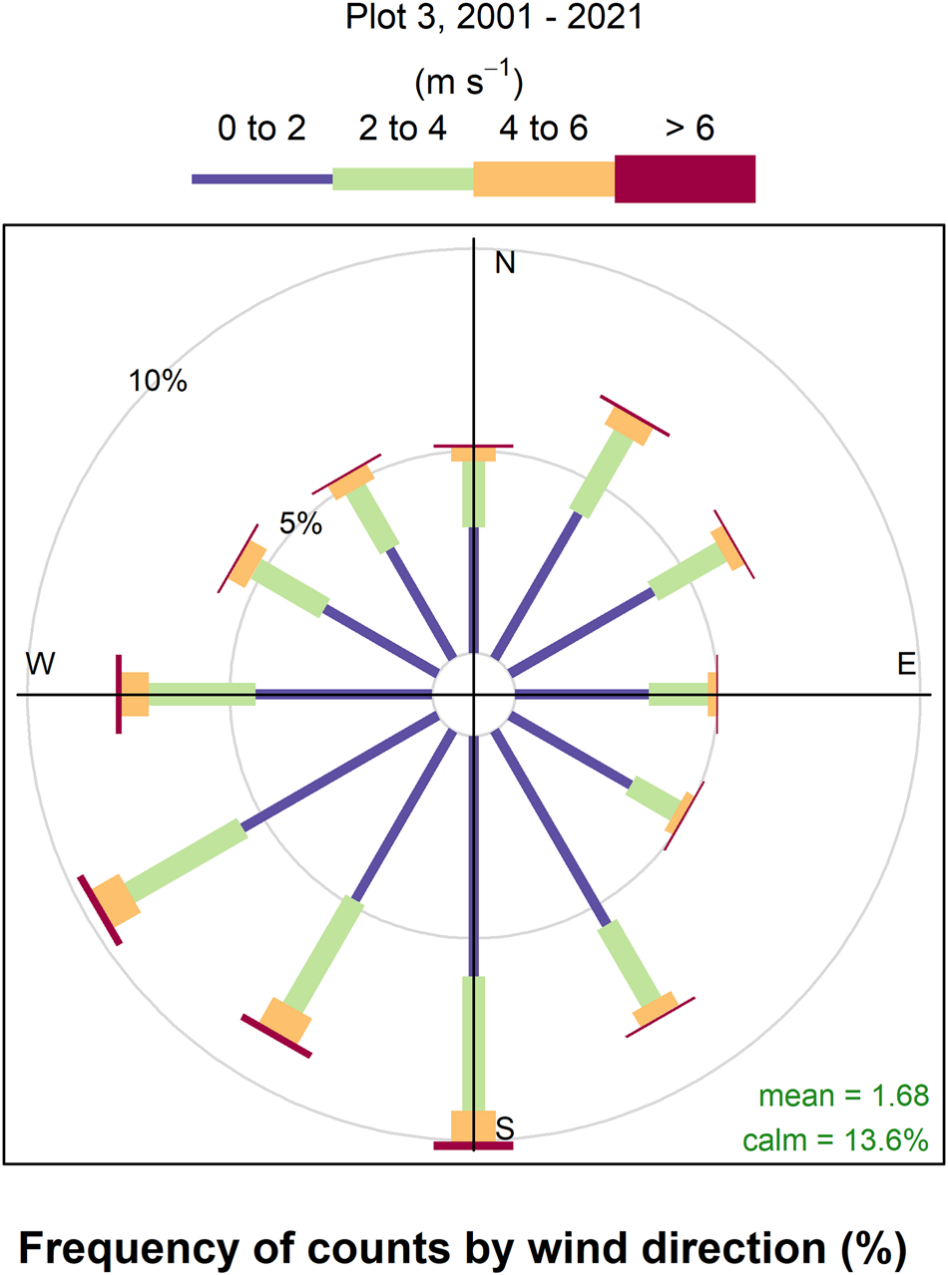
Wind rose plot generated from 1-minute wind speed and direction data collected from Plot 3 between 2001 and 2021. Wind rose plots are polar graphs generated with the *openair* package in R^48^. The radii length of the concentric circles represents the percentage frequency of measurements that have wind speeds between 0 to 2 m/s (blue), 2 to 4 m/s (green), 4 to 6 m/s (orange), and >6 m/s (red). The wind direction ranges include plus or minus 15 degrees from the given direction, starting at 0° on the upper vertical axis (N) and moving clockwise in 30° increments back to 360°. In the lower right corner of the plots, the *mean* refers to the overall mean wind speed for the plot, and the *calm* percentage indicates the percentage of calm observations omitted from the wind rose plot. Following the convention of the National Weather Service, winds with a direction of 0° are considered ‘calm’, while winds with a direction of 360° are assumed to be from the north.

Wind serves a crucial role in the fumigation experiments by dispersing CO_2_ and O_3_ throughout each treatment plot, and therefore accurate wind data are important. To this end, wind data for each plot are recorded at 4-second intervals along with the CO_2_ and O_3_ fumigation levels, setpoint gas levels, and flow valve settings. In particular the wind direction data have been analyzed at SoyFACE, with the prevailing wind direction at the experiment site found to be South/Southwest with some variation between treatment plots due to the difference in location (Figs. 2, 3). The central computer at SoyFACE receives the 4-second and 1-minute fumigation data files (which are averaged from the 4-second data files), and stores both sets of data. Occasionally, there have been data losses and errors at SoyFACE due to extreme weather or technological issues with the analyzers or computer systems. The use of customized Matlab computer code and functions (detailed in the *Matlab Files* sub-section of the *Data Records* section) allows the identification of gas measurements that are outside the expected threshold, or ‘filter window’, along with possibly erroneous repeated values. The filter window for [CO_2_] was determined to be 250–1500 ppm, while the filter window for [O_3_] was 0–500 parts per billion (ppb). After the processing and quality control of the 1-minute fumigation data files, the fumigation measurements were averaged (i.e., the mean and median values were calculated from the 60 1-minute measurements over each hour) via computer code to produce hourly fumigation files^37^ (File 11). The hourly fumigation files have proven to be useful, as they are more commonly used in plant growth models than the 4-second and 1-minute fumigation data files. Ambient [CO_2_] and [O_3_] are measured at a central location in the SoyFACE field and stored in 1-minute ambient files in the central computer, which have also been consolidated into hourly files.

Precipitation and solar radiation data were recorded at the Water and Atmospheric Resources Monitoring (WARM) station in Champaign (https://warm.isws.illinois.edu/warm/), while other weather metrics such as temperature, wind speed, and relative humidity were recorded at the Surface Radiation (SURFRAD) station about 16 km southwest of the SoyFACE farm (https://gml.noaa.gov/grad/surfrad/bondvill.html). Collectively, the WARM and SURFRAD meteorological data are referred to as CMI weather data due to the stations’ proximity to the University of Illinois-Willard Airport (CMI). Computer code that is publicly available on GitHub (https://github.com/eloch216/oscillator-based-circadian-clock-analysis/) can be used to process and consolidate the CMI weather data collected from the WARM and SURFRAD stations into hourly files from 1995 through 2021. These hourly CMI weather data files have been combined with hourly ambient [CO_2_] and [O_3_] data into ‘Hourly Weather and Ambient Data’ files^37^ (File 6), which provide a summary of the atmospheric and meteorological conditions for each year of the SoyFACE experiments.

Additional useful metrics include the O_3_ exposure indices AOT40, SUM06, and W126 (Eqs. 2–4), which are included in the ‘SoyFACE Hourly Fumigation Data’ files. In these equations, *i* = *1, …, n* represent the daylight hours between 8:00 AM and 7:00 PM. The O_3_ exposure indices were measured in parts per billion initially and then converted to parts per million. Unlike the AOT40 and SUM06 indices, which ignore all O_3_ concentrations below a certain threshold, the W126 index is a sigmoidal weighted function that gives preferential treatment to higher concentrations of O_3_ up to 100 ppb (0.1 ppm) without ignoring the lower concentrations^40^. All three O_3_ indices measure the accumulated daytime O_3_ exposure throughout the entire growing season.

2 $$AOT40={\sum }_{i=1}^{n}({[{O}_{3}]}_{i}-40),\;for\;{[{O}_{3}]}_{i} > 40\,ppb$$

3 $$SUM06={\sum }_{i=1}^{n}{\left[{O}_{3}\right]}_{i},\;for\;{\left[{O}_{3}\right]}_{i} > 60\,ppb$$

4 $$W126={\sum }_{i=1}^{n}{\left[{O}_{3}\right]}_{i}\cdot \frac{1}{\left(1+4403\cdot {e}^{-126\cdot {\left[{O}_{3}\right]}_{i}}\right)}$$

Another important metric that can be computed from the SoyFACE experimental data is the fumigation target percentages (Table 3). This metric calculates the proportion of minutes that the CO_2_ or O_3_ level is within 10% or 20% of the setpoint when the fumigation system is turned on, which provides insight into the efficiency and accuracy of the SoyFACE experiments. These data can also be found in the ‘Fumigation Target Percentages’ file; the code that generates the data is described in the supplementary explanatory file^37^ (Files 2-3).

**Table 3:** Fumigation efficiency data for SoyFACE experiments between 2001 and 2021.

Year	10% Target CO2	20% Target CO2	10% Target O3	20% Target O3
2001	81.5	92.5	N/A	N/A
2002	73.7	86.2	74.9	89.2
2003	84.7	94.0	80.7	95.2
2004	84.6	95.3	80.9	95.0
2005	81.9	93.0	78.3	94.1
2006	85.0	95.1	82.5	95.7
2007	82.0	93.2	73.4	91.7
2008	82.9	94.0	68.2	87.9
2009	75.7	89.8	57.4	80.3
2010	65.5	87.1	52.2	72.7
2011	78.6	91.2	73.2	90.7
2012	75.6	90.2	71.5	88.2
2013	74.5	89.3	56.6	77.3
2014	78.3	92.0	59.0	82.4
2015	82.3	93.0	55.8	79.4
2016	83.6	94.1	65.6	87.4
2017	85.3	96.0	60.2	82.9
2018	87.9	96.6	61.0	84.0
2019	85.1	95.9	62.5	85.3
2020	82.4	93.8	60.9	83.9
2021	79.4	92.6	63.4	84.2

Percentages are calculated by dividing the total amount of minutes that the fumigation system is turned on and the CO_2_/O_3_ measurement is within 10% or 20% of the setpoint by the total amount of minutes that the fumigation system is turned on. Note that overall, the CO_2_ fumigation process is more precise than the O_3_ fumigation process, maintaining 20% accuracy at least 80% of the time for all years of the CO_2_ experiments. While less precise, the O_3_ fumigation process maintained 20% accuracy at least 80% of the time for 17 out of 20 years of O_3_ experiments, showing a fairly high precision level.

## Data Records

The data records cited in this work are stored in the Illinois Data Bank, which is a public access repository for publishing research data from the University of Illinois at Urbana-Champaign (https://databank.illinois.edu/). This data set consists of 8 files and 4 zipped folders^37^. Descriptions of these data records are as follows.

### SoyFACE plot information 2001–2021 File

This file describes the SoyFACE experiments between 2001 and 2021, including the fumigation treatment type (CO_2_, O_3_, or a combination treatment), crop species, the plot (also referred to as ‘ring’) numbers used for each experiment, planting, treatment, and harvesting dates, and the gas setpoints^37^ (File 12). The full data are also contained in Tables 1–2.

### SoyFACE 1-minute fumigation data files

The raw fumigation data at SoyFACE are initially recorded as 4-second data files, and subsequently averaged to create 1-minute data files. The 1-minute raw data have been quality controlled to remove erroneous repeated values (*Data_Issues_Finder* custom code^37^ (Files 1 and 9).

The quality controlled 1-minute data files are named ‘Avg_MMDDYY’ and contained in the ‘SoyFACE 1-Minute Fumigation Data Files’ folder. The ‘SoyFACE 1-Minute Fumigation Data Explanation’ file contains the column descriptions, units of measurement, and other important notes^37^ (File 8).

### SoyFACE hourly fumigation data files

The hourly SoyFACE fumigation files are generated by averaging the CO_2_ and O_3_ fumigation data from the quality controlled 1-minute data files, ignoring values outside the filter window as described in the *Data Collection & Processing Methods* sub-section. The hourly fumigation files also include ozone exposure metrics AOT40, SUM06, and W126.

The hourly fumigation files are named ‘YYYY_HrlyFumData_ByRing’ and contained in the ‘SoyFACE Hourly Fumigation Data Files’ folder. The ‘SoyFACE Hourly Fumigation Data Explanation’ file contains the formulas for ozone exposure indices AOT40, SUM06, and W126, details about the custom code used to create the hourly fumigation files, and column descriptions for the files^37^ (File 10).

### Hourly weather and ambient data files

The 1-minute ambient CO_2_ and O_3_ data are used to generate hourly ambient data files using the same methods that generate the hourly fumigation data files. The hourly weather data collected from the WARM and SURFRAD stations are combined with the hourly ambient SoyFACE data into single files for each year of the experiments.

The hourly weather and ambient data files are named ‘YYYY_HrlyWeatherData’ and contained in the ‘Hourly Weather and Ambient Data Files’ folder. The ‘Hourly Weather and Ambient Data Explanation’ file contains the column descriptions, units of measurement, and other important notes^37^ (File 4).

### Fumigation target percentages file

The target fumigation percentages file shows the proportion of minutes during each growing season that the fumigation CO_2_ and O_3_ levels are within 10% and 20% of the target concentrations (setpoints) for the SoyFACE experiment when the fumigation system is turned on. The ‘Fumigation Target Percentages Explanation’ file contains details about the custom code used to create the ‘Fumigation Target Percentages’ file, and column descriptions for the file^37^ (File 2). The full data from this file are also contained in Table 3.

### Matlab files

There are several custom Matlab files^37^ (File 7) that were created to process and quality control the ‘SoyFACE 1-Minute Fumigation Data’ files, and to generate the ‘SoyFACE Hourly Fumigation Data’ and ‘Fumigation Target Percentages’ files, as enumerated below:

1. *rings_for_year*: The *rings_for_year* function takes a specific year as user input and generates a list of the rings (plots) used in that year’s SoyFACE experiments as the output variable.
2. *Data_Issues_Finder*: The *rings_for_year* function must be run prior to running this code; the user inputs a specific year into that function, and the output is stored as a variable. This output variable is then used as an input for the *Data_Issues_Finder* code, which loops through the SoyFACE 1-minute raw data files for the year and identifies fumigation measurements that are potentially erroneous by keeping a record of all values that are repeated from one minute to the next. Once the output file has been generated, the user must use qualitative analysis to determine which fumigation measurements are actually erroneous (by comparing the repeated fumigation values to the ambient CO_2_ and O_3_ concentrations, considering the number of repeats in a row, etc.). Usage details can be found in the ‘Data_Issues_Finder Code Explanation’ file^37^ (File 1).
3. *fum*: The *fum* function stores user input details about a specific fumigation experiment as variables, which can then be accessed by other Matlab functions.
4. *batch*: The *batch* code allows the user to run the *HourlyDataFunction* function in bulk, for all dates within the growing period (5/1 through 10/15).
5. *HourlyDataFunction*: The *HourlyDataFunction* function takes the output from the *fum* function as input, along with additional user-provided input. The function uses these inputs to generate output variables such as the file names of the 1-minute fumigation data files, and also calls the *HourlyData* code so that it does not need to be run separately.
6. *HourlyData*: The *HourlyData* code generates the hourly mean and median fumigation metrics from the quality controlled SoyFACE 1-minute fumigation data files, generating output that is used to create the ‘SoyFACE Hourly Fumigation Data’ files. Values outside of the filter window are ignored in calculations of the hourly mean and median fumigation metrics. Further details about items 3–6 can be found in the ‘SoyFACE Hourly Fumigation Data Explanation’ file.
7. *Target_yearly*: The *rings_for_year* function must be run prior to running this code. The *Target_Yearly* code calculates the proportion of minutes that the CO_2_/O_3_ level is within either 10% or 20% of the setpoint when the fumigation system is turned on, generating output that is used to create the ‘Fumigation Target Percentages’ file. Usage details can be found in the ‘Fumigation Target Percentages Explanation’ file.

## Technical Validation

Technical validation of the fumigation data set was achieved by regular maintenance and calibration of equipment. CO_2_ analyzers were calibrated before the start of each growing season and regularly throughout the season using certified gases from an ISO/IEC 17025:2017 accredited source per the manufacturer’s instruction. CO_2_ calibration is at a single point, 750 ppm, and a verification of 0 ppm. The analyzers self-zero approximately every hour. Ozone analyzers were calibrated before the start of the season with a Thermo Scientific 49 C PS or Thermo Scientific 49i PS ozone transfer standard. The standard was either verified by the United States Environmental Protection Agency as a ‘Level 2’ standard or verified by Illinois Environmental Protection Agency as a ‘Level 3’ standard^41^. The US EPA ozone verification program is part of a larger program managed by the National Institute for Standard and Technology.

Ozone analyzers were calibrated during the season, using linear regression at 0 ppb, 50 ppb, 100 ppb, 150 ppb, and 200 ppb. Ozone generators, compressors, and pressure regulators were serviced each field season according to their manuals, and parts were replaced as needed. Data from each of the fumigation plots were compared to test for outliers and the need to calibrate sensors. Wind sensors were maintained each field season and calibrated as needed. The custom software system alerted the FACE site managers of communication and electrical problems.

The fumigation target percentages for each year of the SoyFACE experiments between 2001 and 2021 provide a quantitative measurement of the accuracy of the fumigation process at SoyFACE (Table 3). In particular, Table 3 shows that the CO_2_ fumigation process maintained 20% accuracy (i.e., the measured CO_2_ value was within 20% of the setpoint) for at least 90% of the time for 17 out of 21 years of CO_2_ experiments and maintained 20% accuracy at least 80% of the time for all years. The O_3_ fumigation process maintained 20% accuracy at least 90% of the time for 6 out of 20 years of O_3_ experiments, at least 80% of the time for 17 out of 20 years, and at least 70% of the time for all years. It is clear that the CO_2_ fumigation process is able to achieve greater precision than the O_3_ fumigation process, likely because the target elevated concentration for CO_2_ was an approximate 50% increase over ambient, whereas the elevated O_3_ concentration in recent years was a 150% increase over ambient.

## Usage Notes

This SoyFACE fumigation data set can be used as an input for studies that aim to model the impacts of atmospheric change on crop productivity at field, landscape, or regional scales^42–45^. For example, a recent semi-mechanistic model of soybean biochemistry and growth was developed using data from a few years of the SoyFACE experiment^45^. Having 20 years of SoyFACE data compiled and accessible could enhance the development and testing of such a model. Jin *et al*.^44^ investigated the interaction of rising [CO_2_] and drought stress on regional soybean production, again using only a few years of data from SoyFACE to parametrize the CO_2_ response. Having the full set of fumigation data from SoyFACE could improve scenario simulations, yielding more useful results. The physiological and agronomic data describing crop responses to elevated [CO_2_] and [O_3_] at SoyFACE have been previously published in both original manuscripts^25,31,32^ and meta-analyses^26,46^ and are available as supplemental files in those studies. Here, for the first time, we provide the complete hourly and seasonal fumigation information for 20 years of SoyFACE experiments.

The fumigation data set can also be used to explicitly test how wind speed, wind direction, and other environmental factors impact the precision and efficiency of fumigation. Recent studies have hypothesized that rapid fluctuations in CO_2_ concentration in FACE experiments may reduce the photosynthetic, growth, and yield response of crops to elevated CO_2_ concentrations^47^. Compiled data from both ambient and elevated [CO_2_] plots at SoyFACE may provide additional data to test that hypothesis, and to identify parts of the PID algorithm that could be altered to improve fumigation accuracy and precision.

## Data Availability

The Matlab version used for this work is MATLAB R2022b. The custom Matlab code and functions used to generate several of the supplementary files associated with this work are described in the *Matlab Files* sub-section of the *Data Records* section. The Matlab code and functions are described in further detail in the ‘Explanation’ files, which are publicly accessible via the Illinois Data Bank^37^. The Matlab code and functions are contained in the ‘Matlab Files’ folder (File 7), and the underlying data set is contained in the ‘SoyFACE 1-Minute Fumigation Data Files’ folder (File 9). The R statistical programming language and *openair* package are required in order to use the *windRose* function (Figs. 2, 3). The R version used for this work is Rx64 4.1.2, which is free for all users to download. The underlying data set is contained in the ‘SoyFACE 1-Minute Fumigation Data Files’ folder (File 9).
